# The type II secretion system utilizes GspN for transport of lipoproteins to the *Acinetobacter baumannii* cell surface

**DOI:** 10.1128/mbio.03449-25

**Published:** 2025-12-16

**Authors:** Cameron S. Roberts, Colby Gura, Maria Sandkvist

**Affiliations:** 1 Department of Microbiology and Immunology, University of Michigan Medical School 12266 , Ann Arbor, Michigan, USA; University of Georgia, Athens, Georgia, USA

**Keywords:** lipoproteins, protein secretion, cell envelope, Gram-negative bacteria, *Acinetobacter baumannii*, repeating beta-groove domain

## Abstract

Gram-negative bacteria employ the type II secretion system (T2SS) to not only secrete an array of soluble effectors, such as toxins, to the extracellular space, but also to facilitate the surface localization of enzymes and adhesins that are beneficial to life in different environments. For example, the pullulan-degrading enzyme pullulanase (PulA) from *Klebsiella pneumoniae* and the recently discovered adhesin InvL from *Acinetobacter baumannii* are initially expressed with a lipobox containing a signal peptide, resulting in their N-terminal acylation and subsequent surface anchoring after T2SS-mediated export. While outer membrane translocation of both soluble and surface-associated T2SS effectors depends on the T2SS secretin GspD, it is unclear how lipoproteins are accommodated by the T2SS during transport to the cell surface. Here, we identify a role for GspN in the outer membrane translocation of InvL in the opportunistic pathogen *A. baumannii*. Additional putative lipoproteins are found to have a similar GspN dependence for outer membrane translocation, while soluble proteins are extracellularly secreted in the absence of GspN. We demonstrate that a specific sorting motif C-terminal to the lipobox is required for GspN-dependent surface localization. Based on structural predictions, GspN is homologous to members of the AsmA-like protein family that includes both eukaryotic and prokaryotic proteins containing concave β-taco folds. Although this protein family has been implicated in phospholipid transport, here we show a role for a β-taco fold protein in the transport of lipoproteins. We also confirm that the GspN homolog PulN is required for PulA surface localization in *K. pneumoniae*.

**IMPORTANCE:**

The type II secretion system (T2SS) is considered a virulence factor of Gram-negative pathogens, such as *Acinetobacter baumannii*. Despite a described function for the majority of the core components of the T2SS, the role of GspN has been unclear, and it was previously reported that GspN is dispensable for protein secretion by this system. Here, we characterize the selective transport of a subset of proteins by the T2SS and show that GspN is required for their outer membrane translocation and surface localization.

## INTRODUCTION

The type II secretion system (T2SS) is responsible for the secretion of a variety of effector molecules to the extracellular space in select Gram-negative bacteria (1–3), including proteases, lipases, carbohydrate active enzymes, and toxins, that support environmental persistence and host colonization (2, 4, 5). While many effector molecules are freely released to the extracellular space following outer membrane translocation, several are retained on the bacterial cell surface (1, 6). Cell surface-associated effectors include PulA from *Klebsiella pneumoniae*, heat-labile enterotoxin from *Escherichia coli*, PnlH from *Dickeya dadantii*, VesB from *Vibrio cholerae*, and InvL from *Acinetobacter baumannii* (7–11). Several distinct mechanisms of surface association exist. For example, VesB is retained on the cell surface via a posttranslational modification of its C-terminus with a glycerophosphoethanolamine-containing moiety (12), and PnlH is produced with a non-cleavable TAT signal peptide, which establishes surface association (9). In the case of heat-labile enterotoxin, the B subunit of the toxin binds to lipopolysaccharides (10). Finally, PulA and InvL are produced with a signal peptide containing a lipobox, resulting in lipidation at a conserved cysteine residue and processing by the signal peptidase II prior to secretion and surface anchoring (8, 11). Given their hydrophobic moiety, it is unclear how the T2SS can accommodate both lipoproteins and soluble proteins during transport to the extracellular environment.

**Fig 1:**
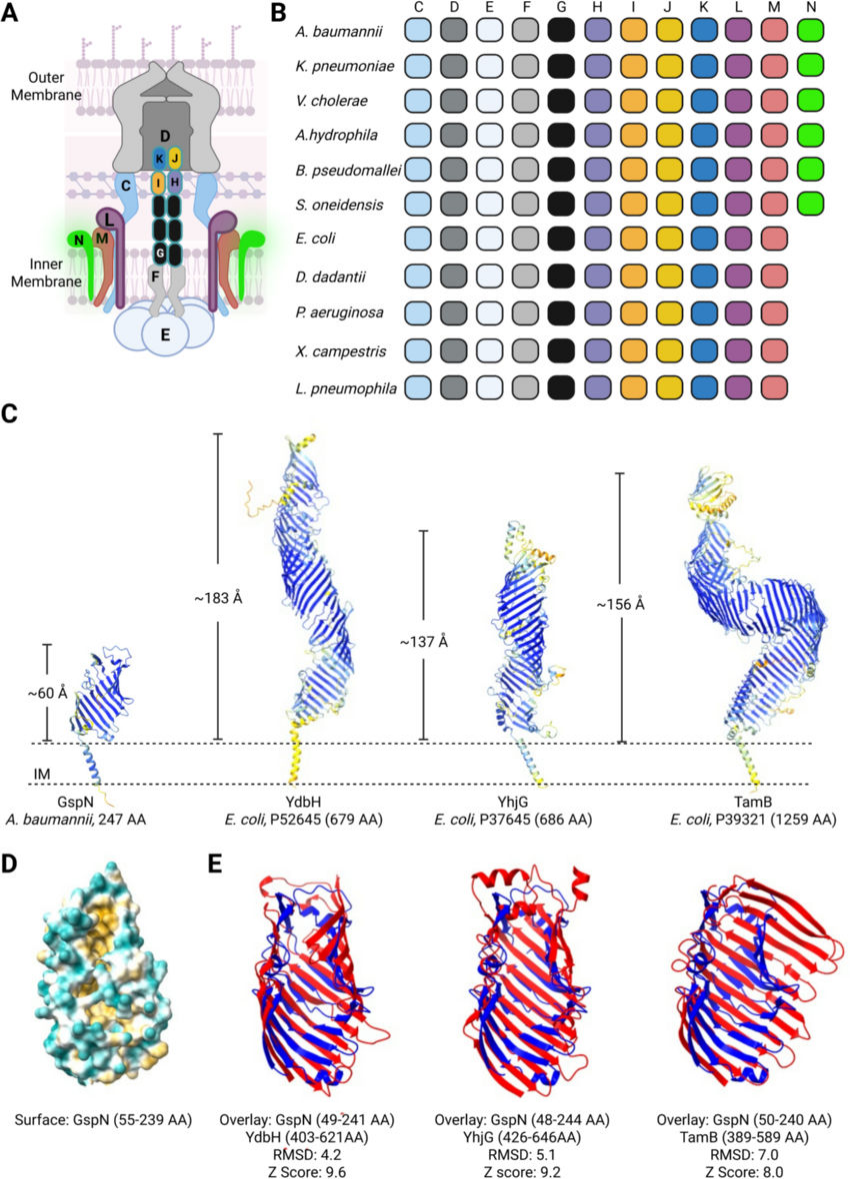
GspN is structurally homologous to AsmA-like proteins. (**A**) Model of the T2SS nanomachine spanning the cell envelope of Gram-negative bacteria. Core components are indicated with letter nomenclature, and a hypothetical position of GspN (in green) is shown. Prepilin peptidase and pilotin are not shown. (**B**) Conservation of genes coding for T2SS core components from representative species. Specific strains analyzed include *gspN*-containing strains *A. baumannii* G414, *K. pneumoniae* KPPR1, *A. hydrophila* ML09-119, *V. cholerae* N16961, *B. pseudomallei* 688, and *S. oneidensis* MR-1, and those lacking *gspN E. coli* E2348/69, *D. dadantii* 3937, *Pseudomonas aeruginosa* PAO1*, Xanthomonas campestris* 85-10, and *Legionella pneumophila* 130b. (**C**) The structure of GspN (encoded by RE180_16620) from *A. baumannii* G414 was predicted using AlphaFold3. The predicted structures of the indicated AsmA-like proteins were extracted from the AlphaFold database. Structures are colored by pLDDT confidence score in dark blue (pLDDT > 90), cyan (70 < pLDDT < 90), and yellow (50 < pLDDT < 70). Aligned residue versus expected error for GspN is shown in Fig. S1. Proteins are manually oriented in the inner membrane (IM). (**D**) Surface representation of the predicted GspN structure is colored by hydrophobicity, with cyan representing the most hydrophilic and gold, the most hydrophobic. (**E**) Overlays of the predicted structure of GspN with YdbH, YhjG, and TamB. RMSD and Z-score for each pair were determined with the Dali server.

The T2SS complex spans the entirety of the cell envelope and is encoded by 13–16 genes often present in a single operon or *gsp* locus (Fig. 1A and B) (1, 13, 14). Classically, each of these genes is considered essential for the function of the entire system, with one notable exception (2, 15, 16). Despite demonstrating the presence of the T2SS gene product GspN in purified secretion complexes from *K. pneumoniae* (14), it has been found to be dispensable for the secretion of effector molecules from this species and in *A. baumannii* (2, 16). While not encoded by all bacteria expressing a T2SS, in addition to *K. pneumoniae* and *A. baumannii*, GspN is also found in, but not limited to, *V. cholerae*, *Burkholderia pseudomallei*, *Shewanella oneidensis*, and *Aeromonas hydrophila* (Fig. 1B) (17). It was previously speculated that GspN may only be needed for the efficient secretion of effector molecules under specific growth conditions or only required for the secretion of a specific subset of effector molecules not yet identified (2).

Here, we probe the requirement for GspN in the secretion of a subset of T2SS effectors that are retained at the cell surface via their acylated N-terminus, such as InvL. These proteins are believed to be posttranslationally modified with two acyl groups by Lgt, before cleavage of their leader peptide by Lsp II, and finally modified with a third acyl group by Lnt based on *E. coli* studies (18). GspN is predicted to adopt a fold similar to the so-called AsmA-like proteins, members of a larger protein family, including prokaryotic and eukaryotic proteins, involved in phospholipid transport (19–23). Using a contemporary clinical isolate of *A. baumannii*, we show that the known T2SS effector InvL, produced with a lipobox, requires GspN for efficient outer membrane translocation and surface localization. We have identified additional T2SS-dependent lipoproteins and validated GspN dependence for several of them. Both GspN and a large number of the T2SS-dependent lipoproteins are highly conserved amongst *A. baumannii* isolates. PulA from *K. pneumoniae* also relies on the GspN homolog PulN for surface localization, and T2SS sorting motifs are present within lipoproteins from *B. pseudomallei* and *S. oneidensis*. Overall, we describe a novel function for GspN.

## RESULTS

### The *gspN* gene is highly conserved among a subset of T2SS-containing bacteria

The T2SS consists of 13–16 proteins that form a complex nanomachine that spans the entire cell envelope (Fig. 1A) (1). This nanomachine is distributed across a subset of Gram-negative bacteria where studies have focused on select model organisms, including *E. coli*, *K. pneumoniae*, *D. dadantii*, *Pseudomonas aeruginosa*, *A. baumannii*, *V. cholerae*, *A. hydrophila, Xanthomonas campestris*, *Legionella pneumophila, B. pseudomallei, and S. oneidensis* (2, 9, 17, 24–29). Of the species listed in Fig. 1B, the structural genes *gspC-gspM* are always present, while *gspN* is restricted to *K. pneumoniae*, *V. cholerae*, *A. baumannii, A. hydrophila, B. pseudomallei, and S. oneidensis*. Given past speculation on the role of, or lack thereof, for GspN in T2SS-mediated protein translocation, we examined the conservation of *gpsN* across three of these species. Of the fully assembled *K. pneumoniae* genomes extracted from NCBI, we identified 3,702 genomes with *gspN* out of 3,703 total analyzed. In the single genome lacking *gspN* (CP110177.1), we identified *gspE*, but none of the other *gsp* genes, suggesting that this strain lacks a functional T2SS. Upon examination of 211 fully assembled *V. cholerae* genomes, nine genomes were found to lack all *gsp* genes, including *gspN*. Of the 882 *A*. *baumannii* genomes, the vast majority (873) contain *gspN*. Those lacking *gspN* are also missing multiple other *gsp* genes. Previously, *A. baumannii* strain AB307-0294 (CP001172.2) has been postulated to lack *gspN* but harbor the other genes required for T2SS function (30). However, our analysis revealed that this strain indeed does have a *gspN* copy whose primary amino acid sequence (ATY45646.1) is highly conserved with GspN from other *A. baumannii* strains. Collectively, our findings suggest that there is evolutionary pressure for species that have a T2SS and *gspN* to conserve said gene. We speculate that *gspN* conservation in these T2SS-containing bacteria implies a specific role for GspN in the transport of a subset of effector proteins.

### GspN is structurally homologous to bacterial AsmA-like proteins

To gain insight into the potential function of GspN, we examined the AlphaFold-predicted structures of the GspN homologs from *A. baumannii*, *K. pneumoniae*, and *V. cholerae. A. baumannii* GspN is predicted with high confidence (Fig. 1C; Fig. S1) to adopt a β-taco fold. Both the GspN homologs, PulN from *K. pneumoniae* and EpsN from *V. cholerae,* are predicted to adopt a similar fold (Fig. S2). Consistent with previous results demonstrating that *K. pneumoniae* PulN co-purifies with the inner membrane complex of the T2SS, consisting of PulC, PulL, and PulM, GspN is predicted to harbor a single alpha helix that could orient the protein in the inner membrane with the β-taco fold facing the periplasmic space. Using the predicted structure of GspN, we used the Dali server to search for similar folds using the predicted structural proteome of *E. coli* as a template. The top three hits with a Z-score ≥8.0, typically used as a cutoff for structural homology (31), and an RMSD ≤7.0 were YdbH, YhjG, and TamB. These three proteins fall into the AsmA-like protein family (19, 21, 22, 32), named for the *E. coli* protein AsmA, and are represented in Fig. 1C. Members of this family carry repeating β-taco folds, concave structures with hydrophobic interiors, that in some cases are predicted to span the entirety of the periplasmic space and are implicated in the transport of phospholipids (20, 32). When we compared the structures of GspN and these proteins, we observed significant similarity within the AsmA-like domain of these proteins (Fig. 1; Fig. S3). Additional proteins with known β-taco folds and similar periplasmic location, such as LptA, LptC, and LptD, from the lipopolysaccharide transport system (33) were also returned as hits with this search, but at much lower Z-scores and RMSD >6.0.

### *A. baumannii* strains encode multiple putative T2SS effectors with a lipobox containing signal peptide

Given the structural similarity between GspN and AsmA-like proteins, we hypothesized that GspN might support the transport of lipoproteins. We therefore searched for lipoproteins in three published data sets on the T2SS-dependent secretomes from *A. baumannii* strains UPAB1, 17978, and AB5075 (11, 30, 34). Using the respective study’s cutoff for putative T2SS-dependent effectors, we identified 16, 5, and 4 lipoproteins in the three respective data sets, amounting to a total of 19 unique lipoproteins across the data sets. Upon compiling a list of putative T2SS-dependent lipoproteins (Table 1), we cross-referenced these proteins against proteomic data sets of outer membrane vesicles (OMVs) produced by *A. baumannii* (35–38). We reasoned that the lipoproteins identified in the T2SS studies are most likely associated with OMVs, given that their acyl groups facilitate membrane interaction. This has previously been demonstrated for the lipoprotein InvL (11). In agreement with this hypothesis, 15 of the 19 identified lipoproteins putatively secreted by the T2SS were also found in the proteomes of OMVs (Table 1). The function of most of these lipoproteins has yet to be determined; however, of the characterized proteins, FilF was previously identified as a cell surface antigen and promising vaccine target (39). The second characterized lipoprotein is InvL, an adhesin that depends on GspD for secretion and surface localization (11). Finally, RE180_13595 has been characterized as a glutamyl transferase and is secreted in a GspD-dependent manner, although the surface localization of this enzyme is still unclear (40).

**TABLE 1:** Verified or putative T2SS substrates with predicted lipobox^*e*^

Locus tag (UPAB1 or 17978)	Locus tags (G414)	Annotation	T2SS substrate citation	OMV citation	Sequence (P1′–P8′)
**D1G37_RS04395**	**RE180_02645 (InvL**)	**Hypothetical protein**	(11)	(11)	**CGGGGDGY**
D1G37_RS10930^*c*^	RE180_14235	Nucleosidase	(11, 34)	(37)	CNDDNSSV
D1G37_RS02935^*c*^	RE180_04040	Glycerophosphodiester phosphodiesterase	(11, 34)	(38)	CNDDDKTE
D1G37_RS16055	RE180_08010	Histidine-type phosphatase	(11)	(35)	CNNNDDQE
D1G37_RS01360	RE180_05685	Alpha/beta hydrolase	(11)	(35)	CGGGSSDD
D1G37_RS11575^*c*^	RE180_13595	Gamma-glutamyltransferase	(11, 34)	(35, 36)	CGDDSSSD
**D1G37_RS10450^*b*^**	**RE180_14780 (FilF**)	**Hypothetical protein**	(11, 30)	(35)	**CGGGSSTI**
**D1G37_RS16605**	**RE180_08535**	**Nucleotidase**	(11)	(35)	**CNDNDDND**
D1G37_RS12710	n/a^*d*^	Alpha/beta hydrolase	(11)	(35)	CGSDNDDT
D1G37_RS08070	RE180_17080	Esterase	(11)	(38)	CNDDNDQD
D1G37_RS00090	n/a^*d*^	Hypothetical protein	(11)		CTGEDGGF
D1G37_RS16985	RE180_08820	Hypothetical protein	(11)		CNDNDHDD
D1G37_RS01565^*b*^^,^^*c*^	RE180_05480	Alpha/beta hydrolase	(11, 30, 34)	(35, 36)	CNDDDDDY
D1G37_RS17565	RE180_09720	Hypothetical protein	(11)	(35)	CGGGSSSG
D1G37_RS12010	RE180_12660	Arylsulfatase	(11)	(38)	CNDNDSQE
**D1G37_RS00315**	**RE180_10730^*a*^**	**Hypothetical protein**	(11)		**CAVFMAGN**
AS1_3831	RE180_03760	Hypothetical protein	(30)	(35)	CGDENSNS
A1S_3745	RE180_09720	Hypothetical protein	(30)	(35)	CGGGSSSG
AS1_3863	n/a^*d*^	Hypothetical protein	(30)		CGGGGDGY

^*a*^ Demonstrated in this study to lack T2SS-dependent secretion.

^*b*^ Also present in strain 17978.

^*c*^ Also present in strain AB5075.

^*d*^ Not encoded by G414.

^*e*^ Bolded proteins studied here.

We next analyzed the conservation of T2SS-dependent lipoprotein genes across *A. baumannii* genomes, sorting the results by international clone (IC) groups (Fig. S4). We excluded RE180_10730 from this analysis for reasons discussed below. Previously, a similar analysis has been conducted for InvL that demonstrated high conservation across the IC groups except for IC1, IC6, and IC8, which we also observed here (11). As for the other lipoproteins, we found that the majority are conserved across all isolates regardless of IC group. For example, FilF is universal amongst all IC groups, besides IC7, where it is still highly conserved. Three lipoproteins, RE180_03760 (IC2 and IC6), A1S_3863 (IC1, IC6 and IC8), and DIG37_RS00090 (IC7), are specific to certain IC groups. Overall, we found that all the strains examined, except for one (CP169766.1), harbor ≥12 lipoproteins. Of the one outliner, this strain harbors the gene product for the putative gamma-glutamyl transferase (RE180_13595), which is universal across all strains examined.

### GspN contributes to the transport of lipoproteins to the extracellular space and is not required for the secretion of CpaA

**Fig 2:**
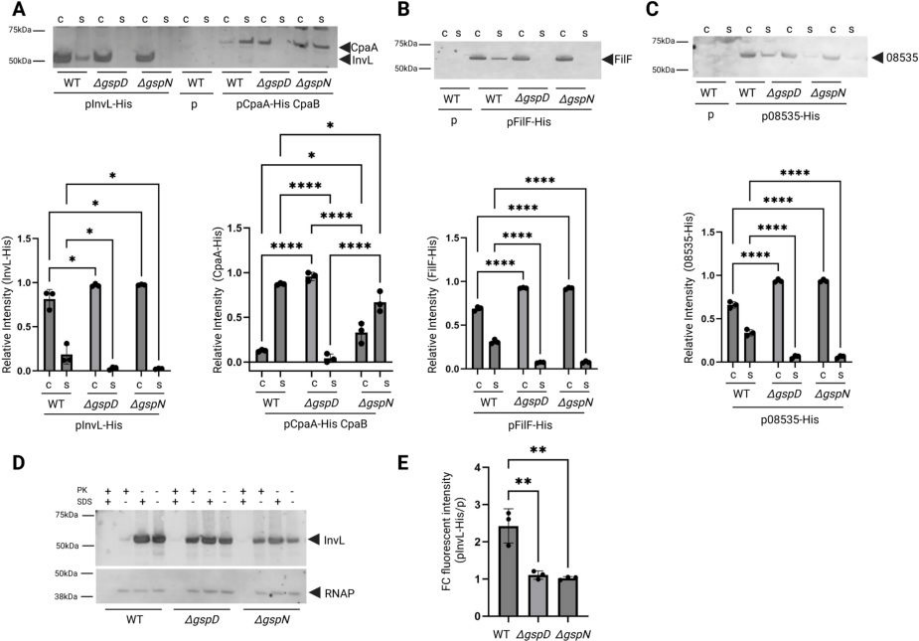
GspN is required for the outer membrane translocation and surface localization of lipoprotein T2SS effectors. (**A**) Overnight cultures from WT and indicated mutant strains containing empty vector (p) or plasmid expressing InvL or CpaA with C-terminal His6 tags were separated into cell (c) and supernatant (s) fractions, run on sodium dodecyl sulfate-polyacrylamide gel electrophoresis (SDS-PAGE), transferred to a nitrocellulose membrane, and blotted with anti-His6 antibody. Positions of molecular mass markers, InvL and CpaA, are indicated. The plasmid encoding CpaA also codes for the chaperone CpaB to ensure proper folding and secretion of CpaA. WT strain with empty vector (p) was used as a negative control. Band intensity from three blots was quantified for InvL-His and CpaA-His with ImageJ, and the percentage of His-tagged protein in the supernatants and cells is plotted below. (**B**) Overnight cultures from WT and indicated mutant strains containing empty vector (p) or plasmid expressing FilF with a C-terminal His6 tag were assessed for FilF secretion as in panel A. Positions of molecular mass markers and FilF are shown. Band intensity from three blots was quantified for FilF-His with ImageJ, and the percentage of His-tagged FilF in the supernatants and cells is plotted below. (**C**) Overnight cultures from WT and indicated mutant strains with empty vector (p) or plasmid coding for 08535 with a C-terminal His6 tag were assessed for 08535 transport as in panel **A**. Positions of molecular mass markers and protein 08535 are indicated. Band intensity from three blots was quantified for 08535-His with ImageJ, and the percentage of His-tagged 08535 in the supernatants and cells is plotted below. (**D**) Cell pellets from overnight cultures from WT and indicated mutant strains expressing InvL with a C-terminal His6 tag were assessed for surface localization of InvL using proteinase K susceptibility with and without permeabilization of cells with 1% SDS. As a control, RNAP blots were stained with antibodies against the RNA polymerase α subunit. Positions of molecular mass markers, InvL, and RNAP are indicated. A representative blot from two biological replicates is shown. (**E**) Cell pellets from panel **D** were used to assess surface localization of InvL by incubating intact cells with His6 antibodies, followed by secondary antibodies conjugated to Alexa Fluor 488 and monitoring the fluorescence intensity using excitation/emission wavelengths of 488/519 nm. Individual values represent relative fluorescence units of the InvL-expressing strain over the strain containing empty vector. Data represent the average of three biological experiments with three technical replicates each ± S.D. For all statistics, one-way ANOVA with Tukey’s multiple comparison test was used with * representing *P* < 0.05, ** <0.005, and **** ≤0.0001. Only significant differences are shown.

Given the ubiquitous nature of the T2SS lipoprotein effectors and GspN in *A. baumannii,* we next sought to experimentally validate a connection between the two. To address this, we first determined if these lipoproteins are encoded by a clinical strain that was recently isolated and sequenced (C. Gura, K. Blair, C. Roberts, and M. Sandkvist, unpublished data). We used SignalP 6.0 prediction (41) to identify 150 putative lipoproteins encoded by G414, which we blasted against the putative T2SS-dependent lipoproteins in Table 1. This revealed that 16 of the 19 identified lipoproteins are encoded by G414. We also identified genes for T2SS core components, including *gspN,* contained within an operon with *gspC* and *gspD*. For our study, we chose to initially focus on the lipoprotein InvL, given its previous characterization and presence in G414. C-terminally histidine-tagged InvL has previously been shown to translocate across the outer membrane to the cell surface in a GspD-dependent manner (11). Subsequent release into the culture supernatant occurs via OMVs (11). To test the potential involvement of GspN in the secretion of InvL, we ectopically expressed InvL with a C-terminal His6 tag in wild-type (WT), ∆*gspD*, and ∆*gspN* mutant strains and examined secretion via western blotting of cell and culture supernatant fractions. As a control, we also analyzed the secretion of the well-characterized and soluble His6-tagged T2SS substrate CpaA (6, 42–46). It should also be noted that we previously showed that the secretion of the T2SS effector LipA, a soluble lipase, is independent of GspN (2). As observed previously, we found a small amount of InvL released into the culture supernatant while the non-lipidated CpaA was completely secreted (Fig. 2). In both cases, however, the release is reliant on GspD. In contrast, only InvL depends on GspN (Fig. 2A). We noted that there is slightly more CpaA in the cell pellet of the ∆*gspN* mutant as compared to the WT strain, but this is in stark contrast to the results observed with CpaA in the ∆*gspD* strain, where all of CpaA is cell-associated. Complementation of the ∆*gspN* mutant with genomically expressed GspN restored the extracellular release of InvL, establishing an essential role for GspN in the transport of InvL but not the soluble protein CpaA (Fig. S5). To verify that the extracellular release of InvL from WT cells is through its association with OMVs, we subjected the culture supernatant from WT cells expressing InvL to sterile filtration and ultracentrifugation to pellet crude OMVs. The majority of InvL co-pelleted at high speed with the lipid content found in the culture supernatant as assessed with the fluorescent dye FM 4-64, confirming that InvL is primarily released from WT cells in the form of OMVs (Fig. S6A). Although a small fraction of InvL was detected in the cleared supernatant following centrifugation, this could be the result of incomplete pelleting of OMVs or through removal of InvL from the cells by an unidentified lipase or protease.

To determine if GspN is required for the secretion of additional T2SS-dependent lipoproteins, we expressed both FilF and the putative nucleotidase RE180_08535 with a C-terminal His6 tag in WT, ∆*gspD*, and ∆*gspN* mutant strains and examined secretion via western blotting (Fig. 2B and C). A similar banding pattern to InvL was observed for both proteins, where the WT strain was shown to release a relatively small amount of FilF and RE180_08535, but the ∆*gspD* and ∆*gspN* mutant strains were not. Given the association with OMVs for FilF and RE180_08535 (11, 35), we also confirmed their association with OMVs through high-speed centrifugation and western blotting of soluble and pelleted fractions (Fig. S6B and C). Taken together, these results indicate that GspN is required for the release of a subset of T2SS effectors to the extracellular space of *A. baumannii*.

### GspN is required for surface localization of InvL

We noted that in the case of InvL, FilF, and RE180_08535, the majority of the protein is present in the cell pellet when ectopically expressed even in the WT strain. Previously, this fraction of InvL has been shown to be surface localized (11). To determine if GspN is required to direct InvL to the cell surface, we subjected intact cells of WT, ∆*gspD*, and ∆*gspN* mutant strains expressing InvL to proteinase K accessibility. We observed that for the WT strain, InvL is largely surface localized given its sensitivity to proteinase K digestion in the absence of the permeabilizing reagent sodium dodecyl sulfate (SDS) (Fig. 2D). In agreement with previously published findings, InvL is protected from proteinase K degradation in the ∆*gspD* mutant strain in the absence of SDS (11). A similar banding pattern to the ∆*gspD* mutant was observed for InvL expressed in the ∆*gspN* strain, indicating that GspN is required for the surface localization of InvL. To verify the role of GspN, we performed the same experiment with the *gspN* complemented ∆*gspN* strain, and again, observed near complete proteinase K susceptibility in the absence of SDS (Fig. S5B). Using a quantitative approach, we also subjected intact cells to antibody labeling and measured the fluorescence intensity against His6-tagged InvL using anti-His6 antibodies and Alexa Fluor 488 conjugated secondary antibodies (Fig. 2E, Table 2). We found that when InvL was expressed in WT cells, we observed significantly higher fluorescence intensity as compared to ∆*gspD* and ∆*gspN* mutant cells expressing InvL in agreement with our proteinase K sensitivity-based results.

**TABLE 2:** Relative fluorescence intensity of cells containing indicated plasmid over cells containing empty vector as measured against His6-tag with anti-His6 and Alexa Fluor 488 secondary antibodies^*a*^

	WT	∆*gspD*	∆*gspN*
pInvL	2.42 ± 0.37	1.10 ± 0.09	1.02 ± 0.03
pSP^Blac^-InvL	0.97 ± 0.07	0.94 ± 0.04	0.85 ± 0.10
p10730	1.03 ± 0.10	1.02 ± 0.08	0.89 ± 0.05
pSP^Pal^-InvL	1.04 ± 0.07	1.11 ± 0.07	1.04 ± 0.08
pSP^LirL^-InvL	0.92 ± 0.09	0.98 ± 0.05	0.93 ± 0.22
pSP^08535^-InvL	2.16 ± 0.11	0.78 ± 0.04	0.98 ± 0.08
pPal	1.06 ± 0.03	ND^*b*^	ND
pLirL	0.97 ± 0.11	ND	ND
pSP^InvL^-Pal	1.03 ± 0.12	ND	ND

^*a*^ Average ± standard deviation (*n* = 3).

^*b*^ ND indicates not determined.

To determine if GspN-dependent outer membrane translocation and surface localization of InvL requires its lipobox, we generated a chimeric InvL species by replacing its own signal peptide with that of the normally periplasmic protein β-lactamase. First, we used SignalP 6.0 to predict the signal peptides and respective cleavage sites of the β-lactamase and InvL precursors (41). Then we substituted the signal peptide of InvL with the signal peptide from β-lactamase. This His-tagged construct, SP^βlac^-InvL, was expressed in the WT cells and subjected to proteinase K treatment (Fig. S7A). We found that this chimeric protein was protected from proteinase K digestion in the absence of permeabilizing reagent. We also quantitatively validated that SP^βlac^-InvL is not surface localized by performing surface labeling and quantitation using anti-His6 antibodies and Alexa Fluor 488 conjugated secondary antibodies (Table 2). To further confirm the intracellular location and loss of membrane interaction for InvL when expressed in the absence of its native lipobox, we subjected WT cells expressing SP^βlac^-InvL to subcellular fractionation (47). As a control for periplasmic content, we assessed each fraction for β-lactamase activity using nitrocefin as a substrate. SP^βlac^-InvL is primarily associated with the β-lactamase activity-containing periplasmic fraction (Fig. S7B). These findings support the previous conclusion (11) that InvL is indeed a lipoprotein and uses its lipobox for membrane association, and that without the lipobox, InvL is no longer transported to the cell surface by the T2SS.

### A specific sorting motif is required but not sufficient for T2SS-mediated transport of lipoproteins

**Fig 3:**
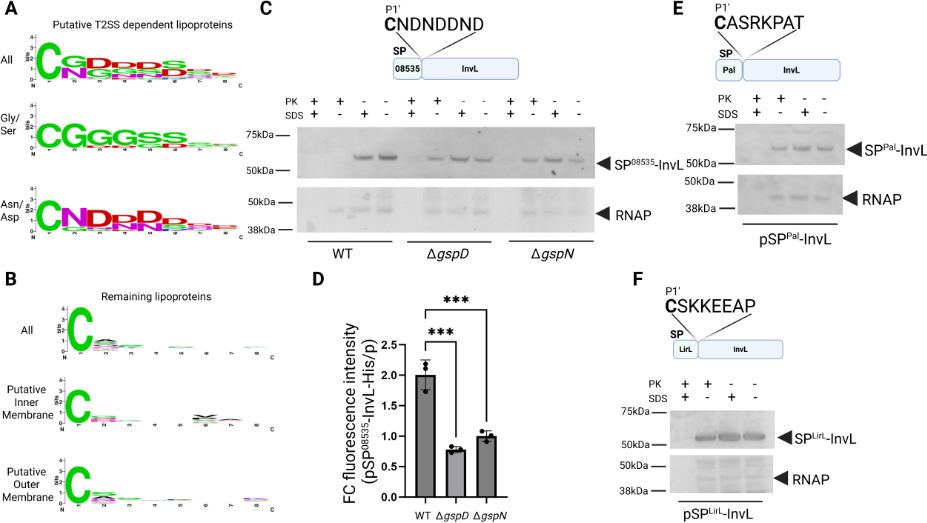
A specific sorting motif is present in lipoprotein T2SS effectors of *A. baumannii* and required for surface localization. (**A**) Sequence logo generated with WebLogo showing residues P1′–P8′ of all putative T2SS-dependent lipoproteins identified in Table 1, with the exception of 10,730. Sequences were further separated into glycine/serine-rich and asparagine/aspartate-rich, with the resulting logos represented. (**B**) A similar sequence logo was generated for the remaining lipoproteins predicted from the G414 genome. Subcellular localization was predicted using Psort to subsequently generate inner membrane and outer membrane sequence logos (list of proteins used can be found in Table S1 and S2, respectively). (**C**) Schematic of signal peptide InvL chimera in which the signal peptide of InvL was replaced with that of 08535 to generate SP^08535^-InvL, where P1′–P8′ residues are represented in relation to the predicted signal peptidase II cleavage site for 08535 (top). Cell pellets from overnight cultures were assessed for surface localization of SP^08535^-InvL using proteinase K susceptibility with and without permeabilization of cells with 1% SDS. As a control, blots were stripped and re-stained with antibodies against RNA polymerase α subunit. Positions of molecular mass markers, SP^08535^-InvL and RNAP, are indicated. Representative western blots (*n* = 2–3). (**D**) Cell pellets from panel C were used to assess surface localization of SP^08535^-InvL by labeling with His6 antibodies, followed by Alexa Fluor 488 conjugated secondary antibodies and monitoring the fluorescence intensity using excitation/emission wavelengths of 488/519 nm. Individual values represent fold change (FC) of relative fluorescence units (RFUs) of SP^08535^-InvL-expressing strain over empty vector containing strain, where an average ± S.D. of three biological experiments was used with three technical replicates. One-way ANOVA with Tukey’s multiple comparison test was used with *** representing *P* < 0.0005. Only significant differences are shown. (**E**) Schematic of signal peptide InvL chimera in which the signal peptide of InvL was replaced with that of Pal to generate SP^Pal^-InvL, where P1′–P8′ residues represented in relation to predicted signal peptidase II cleavage site for Pal are indicated (top). Cell pellets from the WT strain expressing the His6-tagged SP^Pal^-InvL were assessed for surface localization of SP^Pal^-InvL using proteinase K susceptibility with and without permeabilization of cells with 1% SDS. As a control, blots were stripped and re-stained with antibodies against RNA polymerase α subunit. Positions of molecular mass markers, SP^Pal^-InvL and RNAP, are shown. (**F**) Schematic of signal peptide InvL chimera in which the signal peptide of InvL was replaced with that of LirL to generate SP^LirL^-InvL, where P1′–P8′ residues represented in relation to predicted signal peptidase II cleavage site for LirL are indicated (top). Cell pellets from the WT strain expressing the His6-tagged SP^LirL^-InvL were assessed for surface localization of SP^LirL^-InvL using proteinase K susceptibility with and without permeabilization of cells with 1% SDS. As a control, blots were stripped and re-stained with antibodies against RNA polymerase α subunit. Positions of molecular mass markers, SP^Pal^-InvL and RNAP, are shown. In all cases, representative western blots are shown (*n* = 2–3).

Lipoproteins are often sorted to their final destinations in Gram-negative bacteria by a specific sequence C-terminal to the acylated cysteine residue of the lipobox, but sorting rules vary across species (48). We reasoned that lipoproteins destined for T2SS-mediated surface localization might have a unique sequence motif that facilitates their trafficking to the cell surface. Previously, a cell surface signal was identified in the *Bacteroides* phylum that can heterologously target proteins to the cell surface (49), while another distinct signal was identified in proteins enriched in OMVs (50). Using the predicted lipobox sequences of the T2SS effectors encoded by G414 shown in Table 1, we generated a sequence logo (51) starting with the universal cysteine residue at position P1′ relative to the signal peptidase II cleavage site and ending at P8′ (Fig. 3A). This analysis revealed the enrichment of glycine, serine, aspartate, and asparagine residues, which we used as a cutoff to generate chimeric constructs in this study. Manual examination revealed that putative T2SS-dependent lipoproteins are sorted into two groups where members contain either a glycine/serine-rich motif (>4 amino acids) or a motif rich in asparagines/aspartates (>4 amino acids). Sequence logos generated from the sorted groups revealed a strong “GGG” or “ND(N/D)” motif at position P2′–P4′. We next generated a sequence logo for the remaining predicted 134 lipoproteins encoded by G414’s genome at the same positions. This analysis did not reveal any clear sequence motif (Fig. 3B), which may be due to competing sorting motifs between inner and outer membrane localized lipoproteins. To sort any potential distinct motif, we predicted the cellular localization of this group of lipoproteins using Psort (52) yielding 12 proteins predicted to localize to the inner membrane (Table S1), 17 predicted to localize to the outer membrane (Table S2), with the remaining predicted to localize to the extracellular space, periplasmic space, or had a low confidence prediction. Selecting the two membrane subsets to make specific sequence logos again did not result in a clearly recognizable motif at the same position, highlighting the unique signal identified in T2SS-dependent lipoproteins.

One putative T2SS-dependent lipoprotein, RE180_10730, lacks an enrichment of glycine/serine or asparagine/aspartate residues, harboring the sequence “CAVFMAGN.” Given the clear motif present in the remaining putative T2SS-dependent lipoproteins, we chose to follow up on RE180_10730. To assess whether RE180_10730 is indeed transported in a T2SS-dependent manner, we expressed RE180_10730 with a C-terminal His6 tag in WT, ∆*gspD*, and ∆*gspN* mutant strains. We did not observe significant release of this protein to the culture supernatant as determined with western blotting in any of the strains tested (Fig. S8A). We utilized proteinase K susceptibility to rule out that the cellular RE180_10730 is exposed on the cell surface (Fig. S8B) and performed additional validation using surface labeling (Table 2). Furthermore, bioinformatic analysis indicated that RE180_10730 is likely an outer membrane protein that may contribute to the assembly of other outer membrane proteins as it is homologous to SmpA, which forms part of the YaeT (BamA) complex in *E. coli* (53). We therefore speculate that RE180_10730 is a false positive identified in previous secretome studies. Secretome data can easily include proteins present in the inner leaflet of the outer membrane as this group of proteins can also be released through OMVs, although we did not see RE180_10730 in any of the published OMV proteomic data. It is also possible that the expression but not secretion of RE180_10730 is for unknown reasons reduced in T2SS mutants of *A. baumannii*. We note that RE180_10730 was at the lower end of the cutoff threshold in its respective study (11).

After identifying a putative glycine/serine or asparagine/aspartate signal in T2SS-dependent lipoproteins, we tested whether a signal peptide with an adjacent ND(N/D) motif from another GspN-dependent lipoprotein can support the surface localization of InvL which has a GGG motif by generating an InvL chimera consisting of the signal peptide, conserved cysteine, and seven adjacent residues from 08535. Like WT InvL, some SP^08535^-InvL was found in the culture supernatant of the WT strain, but not ∆*gspD* and ∆*gspN* mutants (Fig. S9A). We also verified the surface accessibility of this construct in WT and mutant cells by assessing resistance to proteinase K treatment. We observed that SP^08535^-InvL was sensitive to proteinase K treatment in the WT strain regardless of SDS treatment, while this construct expressed in ∆*gspD* and ∆*gspN* mutants was protected in the absence of SDS (Fig. 3C). In agreement, WT but not mutant cells expressing SP^08535^-InvL were efficiently labeled with His6-tag antibodies and Alexa Fluor 488 secondary antibodies (Fig. 3D, Table 2). Next, we determined if the lipobox from an intracellular lipoprotein would be tolerable for the transport of InvL. Previously, the lipoprotein Pal was identified as an outer membrane protein localized to the inner leaflet (54). To verify that Pal is not a secreted protein, we first expressed Pal with a C-terminal His6 tag in WT, ∆*gspD*, and ∆*gspN* mutant strains, separated cell and supernatant fractions, and probed for Pal via western blotting. We did not observe a significant level of secretion of Pal in any strain despite detection in the cell (Fig. S10A). We also verified that Pal is not exposed on the cell surface of intact cells, as it was resistant to proteinase K treatment and not detected when the cells were incubated with anti-His6 antibodies and secondary antibodies conjugated to Alexa Fluor 488 (Fig. S10B, Table 2). After verifying that Pal is indeed not transported to the cell surface, we generated a chimeric InvL protein, SP^Pal^-InvL, containing the signal peptide, conserved cysteine, and seven adjacent residues from Pal. SP^Pal^-InvL was found primarily associated with the cells and was protected from proteolysis by proteinase K in the absence of permeabilizing reagent (Fig. 3E, Fig. S9B, Table 2). In a similar approach, we generated a second chimera using the signal peptide, conserved cysteine, and seven adjacent residues from LirL, which is a lipoprotein previously found to localize to the inner membrane (55). After validating that LirL was not released to the culture supernatant or surface localized (Fig. S11, Table 2), we probed the transport and surface localization of SP^LirL^-InvL using the proteinase K accessibility assay (Fig. 3F, Fig. S9C, Table 2). We found that SP^LirL^-InvL was primarily associated with cells and was protected from proteolysis in the absence of permeabilizing reagent. Taken together, we conclude that native sorting motifs within the T2SS lipoproteins of *A. baumannii* are permissible for surface localization of T2SS-dependent lipoproteins but not a sequence from the intracellular proteins, such as Pal or LirL.

After verifying that InvL requires a specific motif for outer membrane translocation, we sought to determine if the lipobox from InvL is sufficient to guide a T2SS-independent lipoprotein to the extracellular space. To this end, we generated a chimeric protein consisting of the lipobox, conserved cysteine, and seven adjacent residues from InvL attached to Pal (SP^InvL-^Pal). When we expressed this protein in WT, ∆*gspD*, and ∆*gspN* strains, we primarily observed it in the cell fraction (Fig. S10C). SP^InvL-^Pal in the WT cell fraction was protected from proteinase K in the absence of permeabilizing reagent and was not detected on the cell surface (Fig. S10D, Table 2). We therefore conclude that, in addition to a specific sorting motif adjacent to the lipobox, lipoproteins destined for T2SS-mediated surface localization also require a distinct secretion signal and that the specific sorting motif from InvL is required but not sufficient for surface localization.

### A sorting motif is also present in T2SS-dependent lipoproteins in *B. pseudomallei and S. oneidensis*

Given the presence of GspN homologs in *B. pseudomallei* and *S. oneidensis,* we asked if T2SS-dependent lipoproteins in these species also harbor a sorting motif similar to that of InvL and 08535. Several studies have characterized the functionality of the T2SS in Burkholderia, but a lipoprotein effector has yet to be characterized (56, 57). There are some studies that have characterized the GspD-dependent surface lipoproteins from *S. oneidensis* and showed that they can be used in bioenergy and bioremediation applications (58, 59). Taking advantage of published proteomic data sets from *B. pseudomallei* and *S. oneidensis*, we analyzed the signal peptides of the T2SS-dependent lipoproteins from each of these organisms using SignalP 6.0 (41) (Table S3 and S4). Sequence logos (51) showed a similar sorting motif beginning at the P1′ position relative to the signal peptidase cleavage site as compared to the sequence logo generated from all of the T2SS-dependent lipoproteins from *A. baumannii* (Fig. S12A and B) for both species, albeit the conservation was certainly stronger for *B. pseudomallei*. Manual examination of the individual sequences revealed that most putative T2SS-dependent lipoproteins in these organisms harbor a hybrid motif mostly consisting of glycine and aspartate. This is in contrast to the bifurcated motif found in *A. baumannii*. Given the GspD dependence of these lipoproteins and a conserved sorting motif, we reason that these lipoproteins might also depend on GspN for surface localization.

### Pullulanase depends on PulN for surface localization in *K. pneumoniae*

**Fig 4:**
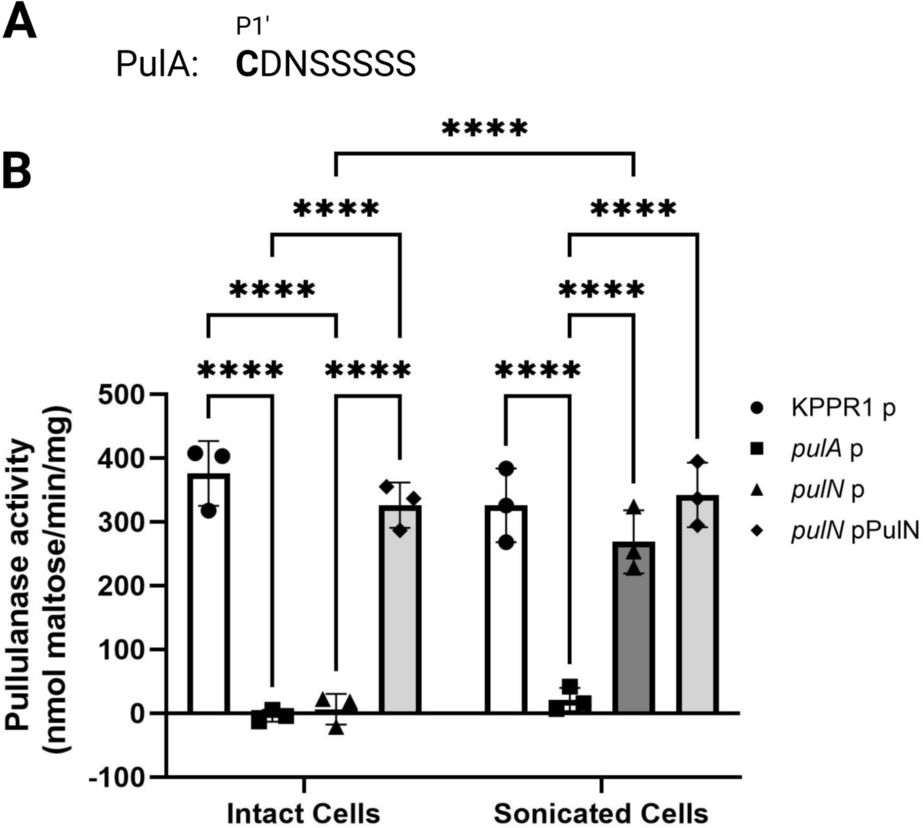
PulN is required for surface localization of pullulanase. (**A**) The sequence starting at P1′ relative to the signal peptidase II cleavage site of the *pulD* (*gspD* homolog) dependent lipoprotein PulA from *K. pneumoniae*. (**B**) Intact and sonicated cells from logarithmic phase cultures of *K. pneumoniae* strain KPPR and mutants with transposon insertions containing empty vector (p) or ectopically expressing PulN (GspN homolog) were isolated and assessed for pullulanase activity by monitoring the reduced sugar content produced over time against a maltose standard curve as determined with 3,5-dinitrosalicylic acid. Experiments were performed in biological triplicates with technical triplicates. Data are presented as mean ± S.D. ****P < 0.0001 by two-way ANOVA analysis with Šídák’s multiple comparisons test. Only significant differences are shown.

Considering our results in *A. baumannii*, we sought to directly establish if T2SS-dependent lipoproteins expressed by other bacteria similarly rely on GspN homologs for secretion. We focused on the lipoprotein PulA that is transported by the T2SS to the cell surface in *K. pneumoniae* and is responsible for the degradation of the polysaccharide pullulan (60). We note that a putative sorting motif C-terminal to the lipobox of PulA slightly differs from that in *A. baumannii* but is enriched in serine residues (Fig. 4A). We assessed the ability of intact cells from WT and two transposon mutant strains with disruption of either *pulA* or *pulN (gspN* homolog) (61) of *K. pneumoniae* KPPR1 to degrade pullulan. Upon incubation of cells with pullulan, we found that the WT and *pulN*::Tn mutant strain complemented with ectopic *pulN* were able to degrade pullulanase as assessed by measuring the rate of reduced sugar generation (Fig. 4B). In contrast, the *pulA*::Tn and *pulN*::Tn mutant strains harboring empty vector had significantly lower pullulanase activity. When the same cells were evaluated for pullulanase activity after sonication, we observed significant pullulanase activity in the WT and *pulN*::Tn mutant strain regardless of plasmid complementation. This demonstrates that PulA is produced but not localized to the cell surface in cells with disruption of *pulN*. Consistent with the role of PulA in pullulan degradation, the *pulA*::Tn mutant strain did not have pullulanase activity even after sonication. These data indicate that PulN facilitates the surface localization of PulA. Collectively, a similar mechanism is suggested to govern the T2SS-dependent transport of lipoproteins to the cell surface across multiple organisms.

## DISCUSSION

In this report, we have demonstrated, for the first time, GspN-dependent secretion of T2SS effectors containing a lipobox with a specific sorting motif in *A. baumannii*. Only some Gram-negative bacteria that encode a T2SS system express GspN, but the gene product is highly conserved in these species, supporting a role in the specialized function in the transport of lipoproteins. Structural prediction reveals a relationship between GspN and members of the AsmA-like protein family known for their roles in phospholipid transfer between the inner and outer membranes in Gram-negative bacteria. The repeating β-taco folds (also called β-groove motifs) of these proteins are thought to shield phospholipids from unfavorable interactions in the aqueous periplasmic compartment (20). In the context of the T2SS, we believe that the hydrophobic β-groove motif of GspN may shield the acyl chains of lipoproteins from unfavorable interactions or extract lipoproteins from the inner membrane, and we have demonstrated a genetic link in support of these hypotheses. Given its likely inner membrane location, we propose that GspN initially recognizes lipoproteins destined for the cell surface after their posttranslational modification. It is possible that additional factor(s) are required for the efficient transport of lipoproteins as opposed to soluble effectors. Additional T2SS components and perhaps other thus far unidentified proteins may aid in transporting lipoproteins, possibly via a hand-off mechanism from GspN. Alternatively, a soluble chaperone similar in function to LolA could be involved (62–64). Other proteins of the T2SS apparatus facilitate specialized functions, such as GspC, that, depending on the species, contains a PDZ domain or a coiled-coil domain that is implicated in extracellular secretion of specific substrates (65–67). Here, we show that GspN has a specialized function in the transport of lipoproteins.

Canonically, the residues adjacent to the acylated cysteine of lipoproteins dictate their subcellular location. For example, in *E. coli,* an aspartate residue is often seen as an inner membrane retention signal (68). However, inner membrane retention signals differ among Gram-negative species (69). In *Pseudomonas*, a “GGG” or “GDD” motif serves as avoidance signals for the localization of the lipoprotein (Lol) pathway, a transport pathway responsible for inserting lipoproteins in the inner leaflet of the outer membrane, promoting inner membrane retention (69). The presence of amino acids serine or lysine also influences sorting in *Pseudomonas* (70). More recently, a unique sorting motif was found in *Bacteroides* lipoproteins that is sufficient for surface localization (49). In our study, bioinformatic analysis revealed the presence of a specific, bifurcated sorting motif in T2SS-dependent lipoproteins of *A. baumannii* that differs from that in intracellular lipoproteins. This sorting motif is required, but not sufficient for outer membrane transport and surface localization of InvL. When the T2SS-dependent lipoprotein InvL was expressed with the corresponding sequence from the normally intracellular Pal or LirL, the resulting constructs were no longer surface localized. Excitingly, similar T2SS sorting motifs are also present in putative T2SS-dependent lipoproteins in other species, and we were able to use this sequence to correctly predict lipoproteins that are not secreted by the T2SS in *A. baumannii*. We speculate that the sorting motif in T2SS-dependent lipoproteins allows both for avoidance of the Lol pathway and efficient recognition by GspN. Previously, PulA from *K. pneumoniae* was found to localize to the inner leaflet of the outer membrane when the aspartate at P2′ was substituted with other residues, promoting recognition by the Lol pathway (71). Another study demonstrated that a purified non-acylated form of PulA can associate with liposomes *in vitro,* and when expressed in *vivo,* it was found to be secreted to the extracellular space by the T2SS (72). In *P. aeruginosa*, it has been proposed that the lipoprotein sorting motif controls the affinity for the inner membrane, promoting either inner membrane retention or outer membrane trafficking (73). These findings, combined with the results here, indicate that the sorting motif in T2SS lipoproteins may promote inner membrane association before recognition by the T2SS. We speculate that, on top of a specific sorting motif, additional structural information within lipoproteins is required for outer membrane translocation. Unlike other systems, such as the Type 9 Secretion System, that recognize an identifiable C-terminal domain in substrates destined for secretion (74), a universal T2SS recognition element has yet to be identified. In contrast, specific structural elements responsible for T2SS recognition have been demonstrated for some effectors, such as VesB from *V. cholerae* (67).

While not all bacteria containing a T2SS carry a *gspN* homolog, both *K. pneumoniae* and *V. cholerae* code for GspN. Seminal work from the Puglsey group identified the genes required for secretion of the lipoprotein PulA from *K. pneumoniae* (3, 15, 16, 71, 75). While initially, PulN was believed to play a role in the biogenesis of PulA (3), it was subsequently shown to be dispensable for PulA secretion in a later study when expressing PulA and the T2SS core components in *E. coli* (16). PulA is thus far the only reported protein to rely on the T2SS in *K. pneumoniae*. While many T2SS substrates have been identified for *V. cholerae,* including the disease-causing cholera toxin, a bona fide lipoprotein T2SS effector has yet to be confirmed. Perhaps additional T2SS-dependent lipoproteins are produced by both these species but primarily localize to the cell surface, explaining how they might have been missed in traditional secretome analyses, especially if their level of expression is low. From the work presented here, we believe a repertoire of surface-associated lipoproteins that previously showed GspD dependence might also depend on GspN for secretion in the notable pathogen *B. pseudomallei*. In addition, *S. oneidensis*, which is being used for biotechnology applications (76, 77), relies on a group of lipoproteins for electron transfer reactions that have previously been shown to depend on GspD for surface localization. We propose that their transport also requires GspN. In *A. baumannii*, T2SS-dependent lipoproteins make up a significant portion of the total number of lipoproteins (13%) and a large portion of the proteins transported by the T2SS. Regarding UPAB1, a urinary tract isolate studied previously, over 50% of the putative T2SS-dependent proteins are lipoproteins (11). Recently, the lipoprotein studied here, InvL, was shown to be important for establishing a chronic respiratory infection (78). Of the few additionally characterized lipoproteins identified in this study to be GspN dependent, the gamma-glutamyl transferase (RE180_13595) has been recognized as a virulence factor (40). These findings demonstrate the importance of further study of this newly identified mechanism of the T2SS.

## MATERIALS AND METHODS

### Bacterial strains and plasmids

The *A. baumannii* strain G414 (CP133499) is a modern clinical isolate from the University of Michigan Hospital. Isogenic mutants Δ*gspD* and Δ*gspN* were generated as previously described (2). The *gspN* complemented strains were generated by overlap extension PCR using genomic G414 DNA and plasmid DNA from pGspN (2) as template with the indicated primers before ligating into pCVD442. The *K. pneumoniae* strain used was KPRR1 and indicated mutant derivatives were generated previously (61). Additional plasmids and primers used in this study are listed in Table S5. All PCR, cloning, and restriction enzyme digestions were done with either SuperFi Platinum polymerase (Fisher) or PfuTurbo (Agilent), T4 DNA ligase (New England Biolabs), and restriction enzymes from New England Biolabs and primers that were synthesized at IDT Technologies. Genes of interest were routinely cloned with the indicated primer using genomic G414 DNA as template before cloning into the Agilent Blunt End Vector Kit (Agilent) per manufacturer’s recommendations and mobilization into pMMB67EH for expression. pMMB67EH constructs were transformed into *E. coli* MC1061 and pCVD442 constructs into SY327λpir. Triparental conjugation was performed with a helper strain carrying pRK2013 to transfer plasmids into G414. Plasmids were mobilized into KPRR1 via electroporation. Indicated primers were used to generate the signal peptide chimeric proteins along with Fwd and Rev pMMB67EH primers, where template DNA constituted plasmid DNA coding for the two proteins of interest before ligation back into pMMB67EH.

### Growth conditions

Strains were grown on Luria-Bertani (LB) agar/broth (Fisher) with 100 µg/mL of carbenicillin (Sigma) when plasmids were present, unless otherwise indicated. IPTG was added at a concentration of 50 µM to induce the expression of InvL and chimeras, CpaA, and Pal. IPTG was omitted from the growth media of strains expressing LirL, FilF, 08535, and 10,730. For the growth of *K. pneumoniae* strains, overnight cultures were started in LB broth, followed by an outgrowth in M9 salts supplemented with 0.4% casamino acids and 0.4% maltose until an OD of 0.9 was reached.

### Sodium dodecyl sulfate-polyacrylamide gel electrophoresis and immunoblotting

Overnight cultures were normalized to an OD 600 of 2.5 before separating cells from supernatant fractions by centrifugation at 10,000 × *g* for 10 min. Fractions were analyzed by sodium dodecyl sulfate-polyacrylamide gel electrophoresis and immunoblotting as described previously. Antibodies against 6-His (Invitrogen, PA1983B) and the α subunit of RNAP (Biolegend, 663903) were incubated with membranes for 2 h (1:5,000). Following washing, horseradish peroxidase-conjugated goat anti-rabbit immunoglobulin G or goat anti-mouse immunoglobulin G (Bio-Rad, Thermo Fisher), used at 1:20,000, was incubated with the membrane for 1 h. Membranes were developed using ECL 2 Western blotting reagent (Thermo Fisher) and visualized using a Typhoon V variable mode imager system and analyzed with ImageJ imager software. Where indicated, band intensity was quantified with ImageJ by measuring the intensity of the band subtracted from background intensity.

For proteinase K accessibility assays, stationary phase cultures were resuspended in LB to an OD at 600 nm of 2.5. Suspensions were treated with 200 μg/mL proteinase K (11) or an equal volume of LB in the presence or absence of 1% SDS at 37°C for 30 min. Phenylmethylsulfonyl fluoride was added at 1 mM to stop the digestion, followed by SDS-PAGE and western blotting analysis as described above.

### Cell surface detection of His6-tagged proteins

Cells were washed, blocked with 2% BSA, and incubated with 1:1,000 of antibody against His6. Following incubation with 1:1,000 of Alexa Fluor 488 F(ab′)2 goat anti-rabbit immunoglobulin G (Invitrogen, A-11070) and washing, fluorescence was measured (Ex 488 nm/Em 525 nm). The results were normalized to the fluorescence intensity of the same strain carrying an empty vector as described (67).

### Crude outer membrane vesicle preparation

Cells were grown overnight before culture supernatants were isolated by centrifugation at 10,000 × *g* for 10 min. Subsequent sterile filtration with 0.2 µm filters and centrifugation at 200,000 × *g* for 3 h pelleted the insoluble fraction. Separately, the cleared supernatant and the pellets containing crude outer membrane vesicles resuspended in LB were assayed via western blotting using 6-His antibody as described above. As a control, the fluorescent dye FM 4-64 (Invitrogen) was incubated with each fraction for 10 min in the dark, before monitoring the fluorescent intensity with excitation/emission wavelengths of 558/734 nm as described (47). Background intensity from media alone was first subtracted before normalizing to the intensity in total supernatant fractions.

### Subcellular fractionation

Log phase WT cells were harvested before resuspending in buffer containing 50 mM Tris, pH 7.5, 100 mM Sucrose, and 1 mM EDTA. Lysozyme was added to a final concentration of 1 mg/mL before incubation for 5 min at room temperature. Samples were centrifuged at 10,000 × *g* for 15 min, and the resulting supernatant was collected as the periplasmic content. The remaining pellet was resuspended in the same buffer with the addition of 1 mM PMSF and 10 µg/mL DNase. Cells were sonicated with a probe-type sonicator. After removing the cell debris, the cytoplasmic and membrane content was separated by ultracentrifugation at 100,000 × *g* for 1 h. The subsequent supernatant fraction was saved as cytoplasmic content, while the pellet was resuspended in the same buffer and taken as membrane content. As a control, β-lactamase activity was assessed with nitrocefin (Toku-E) as described (79). Activity was determined by monitoring the change in absorbance at 500 nm and subtracting the change in absorption of buffer alone. Cytoplasmic, periplasmic, and membrane activities were then added up, and the individual activities were reported as a percentage of the total from the three.

### Assessment of pullulanase activity

Total and surface pullulanase activity was assessed as previously described (80). Briefly, cultures were pelleted, washed three times in phosphate-buffered saline (PBS), before intact cells were resuspended in PBS and incubated with 10 mg/mL pullulan for 4 h. Aliquots were withdrawn at intervals and assessed for reduced sugars using dinitrosalicylic acid against a maltose standard curve. Pullulanase activity is expressed as nanomole maltose produced per minute per milligram of total protein as assessed by Bicinchoninic Acid assay (Thermo Fisher). To compare surface versus trapped pullulanase activity, cells were also sonicated prior to pullulan incubation. As a control, pullulan was incubated with PBS alone. Assays were performed in ≥3 biological replicas in technical triplicates. Two-way ANOVA with Šídák’s correction for multiple comparisons was used to compare all values. Results yielding a *P* value of <0.05 were considered statistically significant; values >0.05 were not shown. All calculations were done using GraphPad Prism version 10.0.0 for Windows (GraphPad Software, Boston, MA, USA; https://www.graphpad.com).

### Structural analysis, sequence alignment, and bioinformatic analysis

The primary amino acid sequence of GspN from G414 was used to predict the secondary structure with AlphaFold3 (81). The predicted structure of PulN and EpsN was downloaded from the AlphaFold database. For structural homology prediction, we used the Dali server (31) to analyze structural homologs to GspN. Structures were visualized with ChimeraX (82). For the sequence logos of G414 lipoproteins, the eight amino acids C-terminal to the signal peptidase II cleavage site were used to generate a logo using the Berkeley WebLogo online tool (51). For the prediction of signal peptides, SignalP 6.0 (41) was used. For gene conservation, complete genomes were extracted from NCBI to form a custom database and blasted against gspN, *pulN*, and *epsN* for *A. baumannii*, *K. pneumoniae*, and *V. cholerae,* respectively, using command line blast. Lipoproteins were similarly blasted against complete *A. baumannii* genomes. Results were subsequently divided into IC groups using a custom script.

All figures were generated with Biorender (BioRender.com).

## SUPPLEMENTAL MATERIAL

Supplemental Information mbio.03449-25-s0001.pdf Figures S1-S12 and Tables S1-S5.

ASM does not own the copyrights to Supplemental Material that may be linked to, or accessed through, an article. The authors have granted ASM a non-exclusive, world-wide license to publish the Supplemental Material files. Please contact the corresponding author directly for reuse.
