# Supplementary material for: The type II secretion system utilizes GspN for transport of lipoproteins to the Acinetobacter baumannii cell surface
Source: mBio. 2025 Dec 16;17(1):e03449-25. doi: 10.1128/mbio.03449-25 (PMC12802150; doi:10.1128/mbio.03449-25)
Supplement: Supplemental Information — Figures S1-S12 and Tables S1-S5. [file mbio.03449-25-s0001.pdf]

Supporting Information

The Type II Secretion System Utilizes GspN for Transport of Lipoproteins to the *Acinetobacter baumannii* Cell Surface

Cameron S. Roberts[1*], Colby Gura[1], Maria Sandkvist[1#]

[1]Department of Microbiology and Immunology, University of Michigan Medical School, Ann Arbor, Michigan, 48109, USA

This PDF includes:

- Figures S1 to S12
- Tables S1 to S5
- SI References

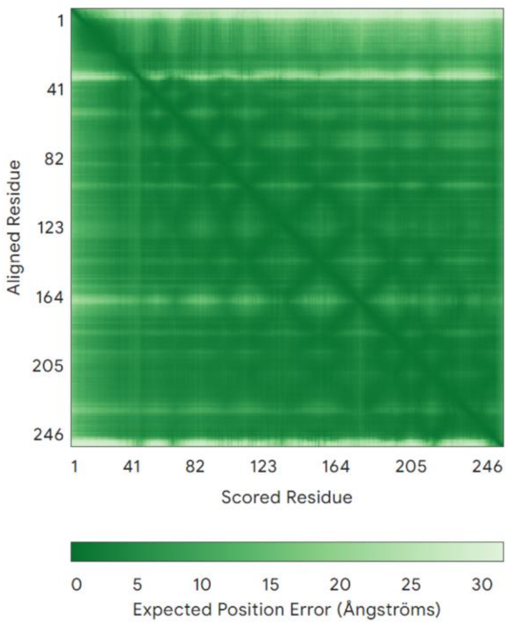

Supplemental Figure 1. Alignment error for *A. baumannii* GspN predicted structure. The primary amino acid sequence of GspN from *A. baumannii* strain G414 was used to predict its structure using AlphaFold3 (1). Predicted error shown.

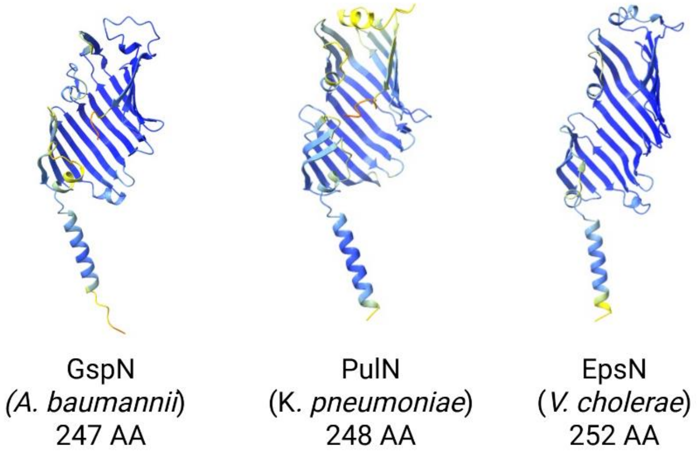

Supplemental Figure 2. Predicted structures of GspN and GspN homologs. The structure of GspN (encoded by RE180_16620) from *A. baumannii* G414 was predicted using AlphaFold3, while the predicted structures of PulN from *K. pneumoniae* (P15753) and EpsN from *V. cholerae* (P45784) were extracted from the AlphaFold database. Structures are colored by pLDDT confidence score in dark blue (pLDDT > 90), cyan (70 < pLDDT < 90) and yellow (50 < pLDDT < 70).

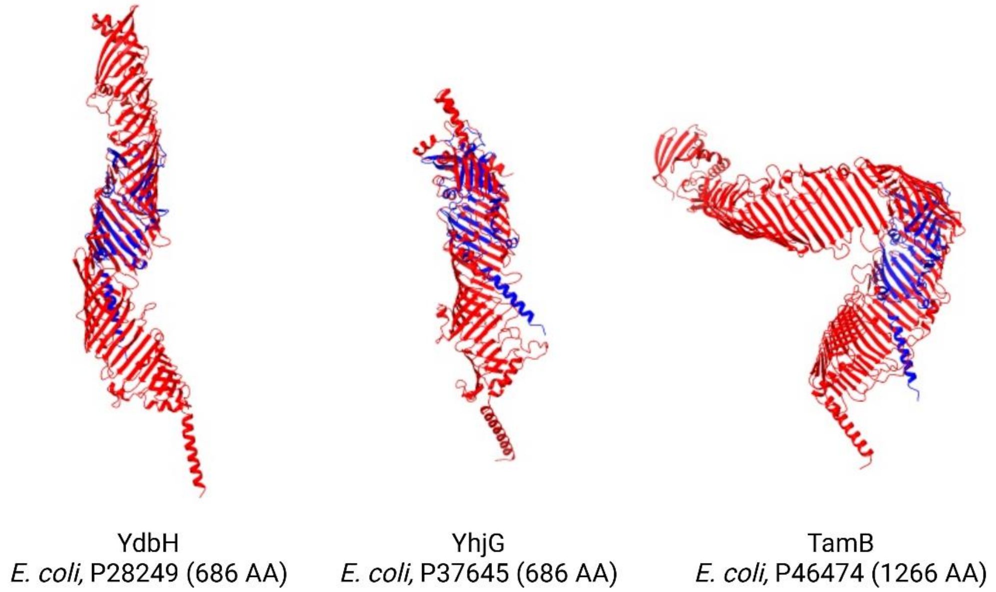

Supplemental Figure 3. Predicted structure of AsmA-like proteins aligned with GspN. The predicted GspN structure was used as query to search the Dali server for homologous proteins. The top three hits are shown with GspN represented in blue and each AsmA-like protein in red. Uniprot and number of residues for each AsmA-like protein is noted.

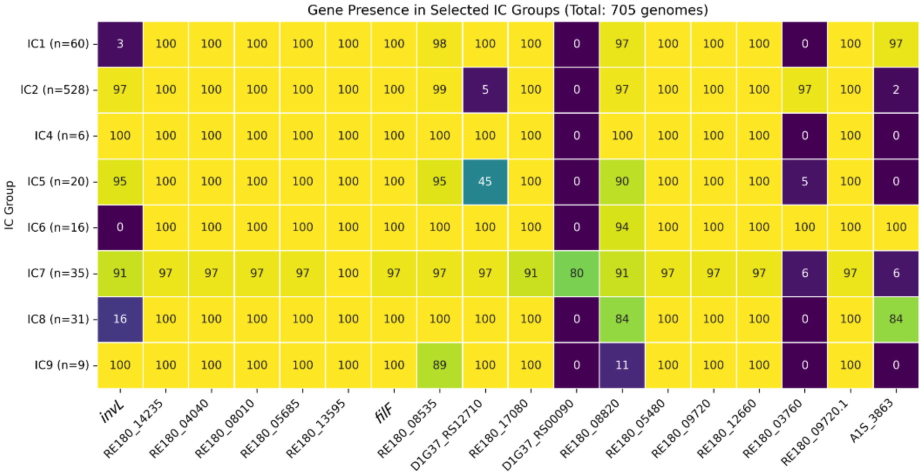

Supplemental Figure 4. T2SS dependent lipoproteins are highly conserved across IC groups. Complete genomes were extracted from NCBI and analyzed for presence or absence of individual lipoprotein genes and shown as percent of total within IC groups. Number of genomes analyzed per IC group is listed.

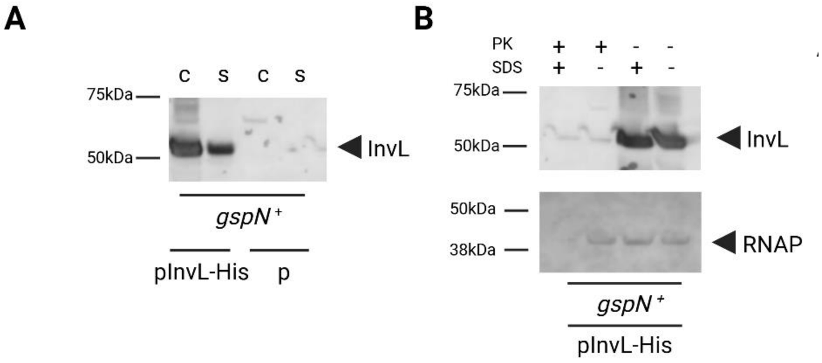

Supplemental Figure 5. Genomic expression of *gspN* restores InvL transport and surface localization. A. Overnight cultures from a *gspN* complemented ∆*gspN* strain (*gspN*[+]) containing empty vector (p) or plasmid expressing InvL with a C-terminal His6 tag were separated into cell (c) and supernatant (s) fractions, run on SDS-PAGE, transferred to a nitrocellulose membrane, and blotted with anti-His6 antibodies. Positions of molecular mass markers and InvL are indicated. **B.** Cell fraction from *gspN*-complemented strain containing pInvL-His6 was assessed for surface localization of InvL using proteinase K susceptibility with and without cell permeabilization with 1% SDS. For a control, RNAP blot was stained with antibodies against RNA polymerase α subunit. Positions of InvL, RNAP and molecular mass markers are shown. Representative western blots are shown (n=3).

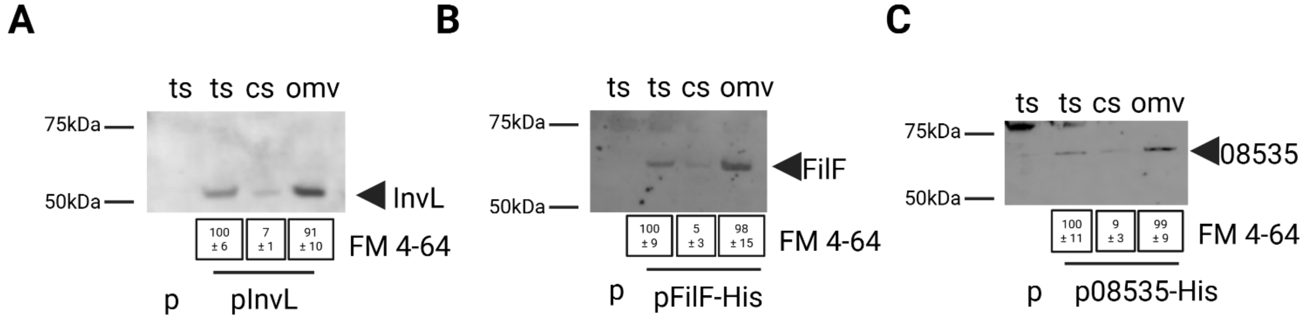

Supplemental Figure 6. T2SS dependent lipoproteins localize in the pelletable fraction of culture supernatant. A. Culture supernatants from overnight cultures of WT strain containing empty vector (p) or plasmid expressing InvL with a C-terminal His6 tag were filter sterilized and subjected to high-speed centrifugation to pellet crude outer membrane vesicles (OMVs) and run on SDS-PAGE along with total supernatants (ts) and cleared supernatants (cs), transferred to a nitrocellulose membrane and blotted with His6 antibodies. As a control, the fluorescence intensity of samples incubated with FM 4-64 was measured to assess lipid content and indicated below the blot as a mean percentage +/- S. D. of ts fluorescence intensity. **B.** Culture supernatants from overnight cultures of WT strain containing empty vector (p) or plasmid coding for FilF were processed and analyzed as in **A**. **C.** Culture supernatants from overnight cultures of WT strain containing empty vector (p) or plasmid expressing 08535 were processed and analyzed as in **A**. Representative western blots are shown (n=2).

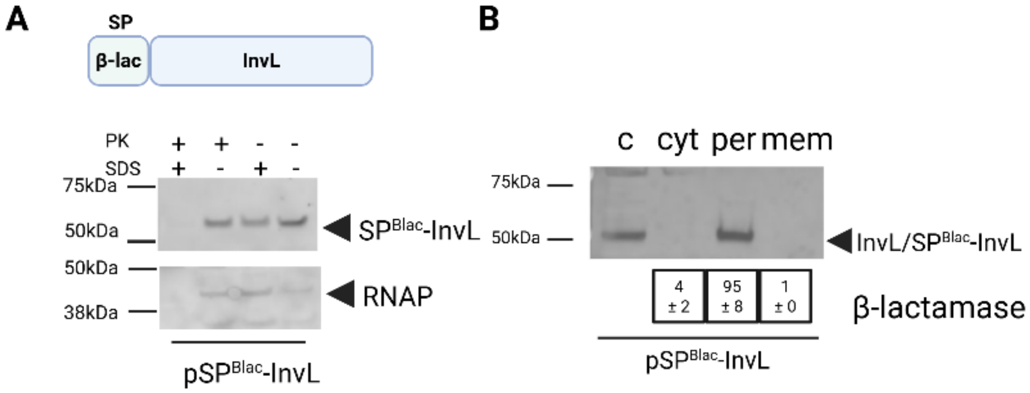

Supplemental Figure 7. InvL produced with the signal peptide from β-lactamase is primarily localized to the periplasm. A. Overnight cultures from a WT strain expressing $SP^{\beta lac}$-InvL were assessed for surface localization of $SP^{\beta lac}$-InvL with proteinase K. For a control, RNAP blot was re-stained with antibodies against RNA polymerase α subunit.Positions of molecular mass markers, $SP^{\beta lac}$-InvL, and RNAP are indicated. **B.** Cell fraction (c) from WT cells expressing SP^{Blac}-InvL were separated into cytoplasmic (cyt), periplasmic (per), and total membrane (mem) before run on SDS-PAGE, transferred to a nitrocellulose membrane, and blotted with anti-His6 antibodies. As a control, β-lactamase activity was assessed with nitrocefin as a marker for periplasmic content. Activity is expressed as mean percentage +/- S. D. of the total activity across the three fractions as described in Materials and Methods.

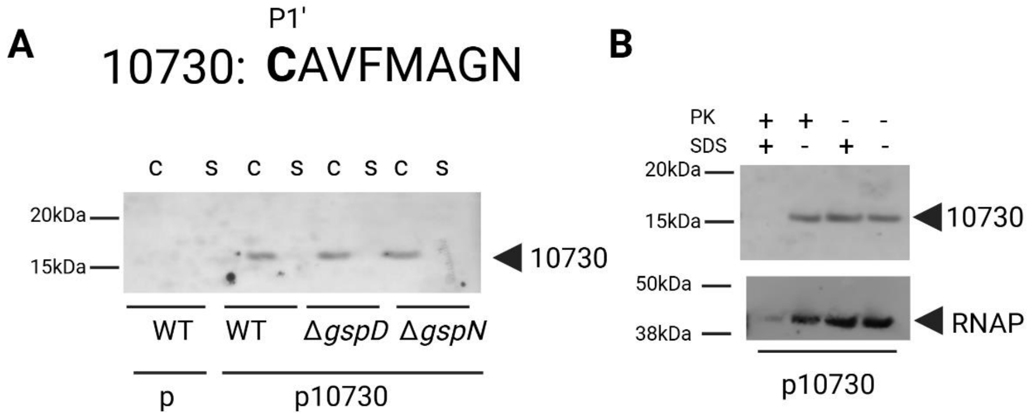

Supplemental Figure 8. 10730 is not secreted by the T2SS in *A. baumannii*. A. P1' – P8' residues represented in relation to putative signal peptidase II cleavage site for 10730 (top). Overnight cultures from WT and indicated mutant strains containing empty vector (p) or expressing plasmid-encoded 10730 with C-terminal His6 tag were separated into cell (c) and supernatant (s) fractions, run on SDS-PAGE, transferred to a nitrocellulose membrane, and blotted with anti-His6 antibodies (bottom). **B.** Cell pellets from overnight cultures from WT and indicated mutant strains containing empty vector (p) or plasmid expressing 10730 with C-terminal His6 tag were assessed for surface localization using proteinase K susceptibility with and without cell permeabilization with 1% SDS. For a control, RNAP blot was re-stained with antibodies against RNA polymerase α subunit. 10730 and RNAP are indicated. Representative western blots are shown (n=2).

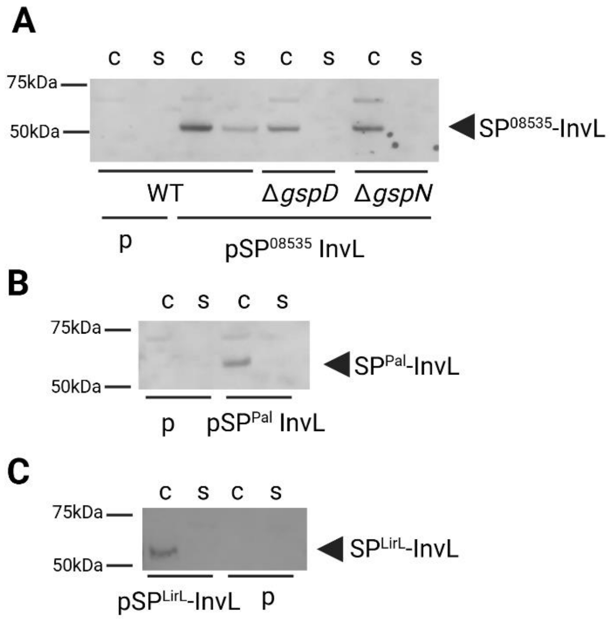

Supplemental Figure 9. A specific sorting motif is required for transport of InvL by the T2SS. A. Overnight cultures from WT and indicated mutant strains containing empty vector (p), or plasmid coding for InvL chimera SP^{08535}-InvL with C-terminal His6 tag were separated into cell (c) and supernatant (s) fractions, run on SDS-PAGE, transferred to a nitrocellulose membrane, and blotted with anti-His6 antibodies (bottom). Positions of SP^{08535}-InvL and molecular mass markers are indicated. **B.** Overnight cultures from WT strain containing empty vector (p) or plasmid coding for the InvL chimera SP^{Pal}-InvL with C-terminal His6 tag were separated into cell (c) and supernatant (s) fractions, run on SDS-PAGE, transferred to a nitrocellulose membrane, and blotted with anti-His6 antibodies. **C.** Overnight cultures from WT strain containing empty vector (p) or plasmid coding for the InvL chimera SP^{LirL}-InvL with C-terminal His6 tag were separated into cell (c) and supernatant (s) fractions, run on SDS-PAGE, transferred to a nitrocellulose membrane, and blotted with anti-His6 antibodies. Representative western blots are shown (n=2-3).

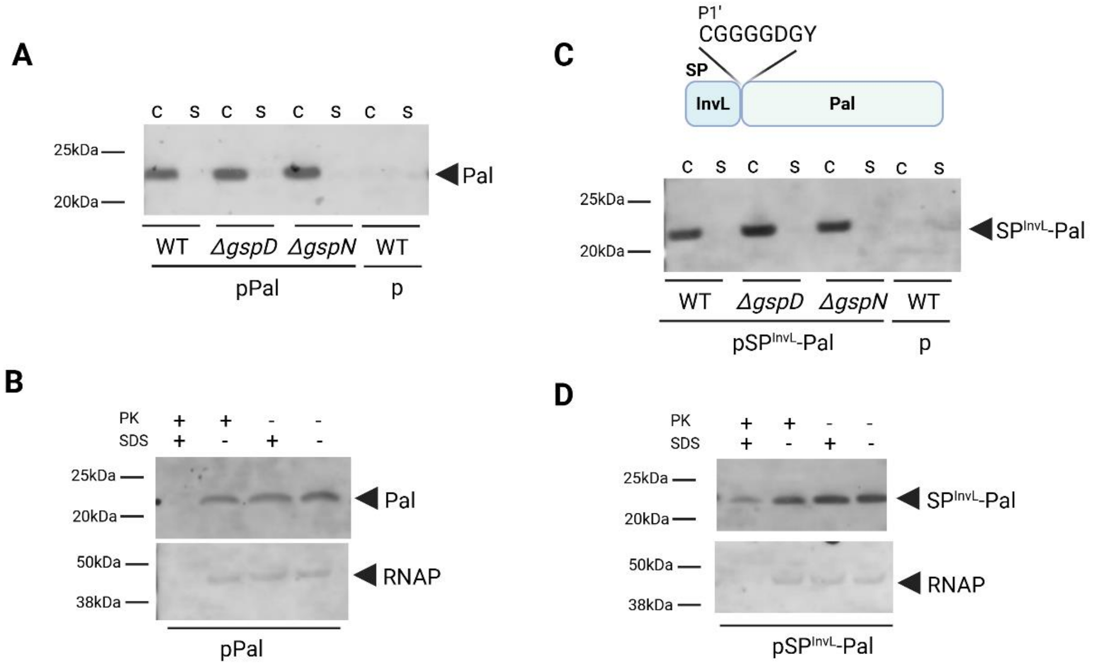

Supplemental Figure 10. The lipobox from InvL is insufficient to target Pal for transport by the T2SS. A. Overnight cultures from WT and indicated mutant strains containing empty vector (p), or plasmid expressing Pal with C-terminal His6 tag were separated into cell (c) and supernatant (s) fractions, run on SDS-PAGE, transferred to a nitrocellulose membrane, and blotted with anti-His6 antibodies. **B.** Cell pellets from overnight cultures from a WT strain expressing Pal with C-terminal His6 tag were assessed for surface localization using proteinase K susceptibility with and without cell permeabilization with 1% SDS. For a control, RNAP blot of the same samples was stained with antibodies against RNA polymerase α subunit. Positions of molecular mass markers, Pal and RNAP are indicated. **C**. Overnight cultures from WT and indicated mutant strains containing empty vector (p), or plasmid expressing chimeric Pal with the signal peptide of InvL (SP^{InvL}-Pal) with C-terminal His6 tag were separated into cell (c) and supernatant (s) fractions, run on SDS-PAGE, transferred to a nitrocellulose membrane, and blotted with anti-His6 antibodies. **D.** Cell pellets from overnight cultures from a WT strain expressing chimeric Pal with the signal peptide from InvL and a C-terminal His6 tag was assessed for surface localization using proteinase K susceptibility with and without permeabilization with 1% SDS. For a control, RNAP blot was stained with antibodies against RNA polymerase α subunit. Positions of molecular mass markers, SP^{InvL}-Pal and RNAP are indicated. Representative western blots are shown from at least two blots performed on biological replicas.

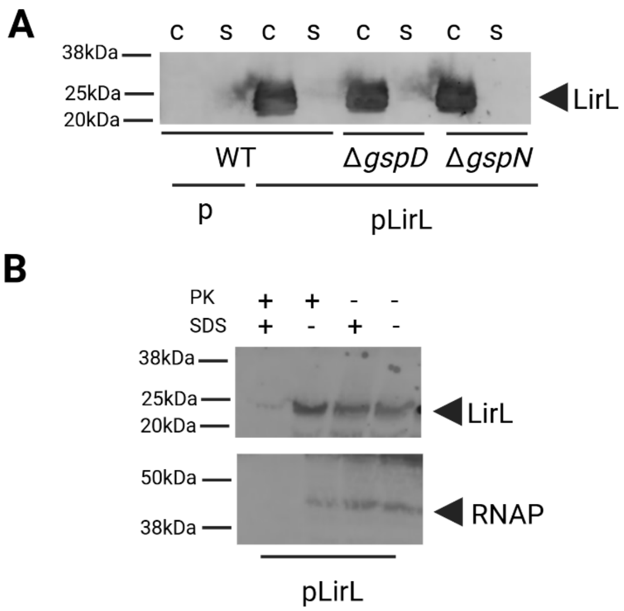

Supplemental Figure 11. LirL is not secreted or surface displayed. A. Overnight cultures from WT and indicated mutant strains containing empty vector (p), or plasmid expressing LirL with C-terminal His6 tag were separated into cell (c) and supernatant (s) fractions, run on SDS-PAGE, transferred to a nitrocellulose membrane, and blotted with anti-His6 antibodies. **B.** Cell pellets from overnight cultures from a WT strain expressing LirL with C-terminal His6 tag were assessed for surface localization using proteinase K susceptibility with and without cell permeabilization with 1% SDS. For a control, RNAP blot of the same samples was stained with antibodies against RNA polymerase α subunit. Positions of molecular mass markers, LirL and RNAP are indicated. Representative western blots are shown (n=2).

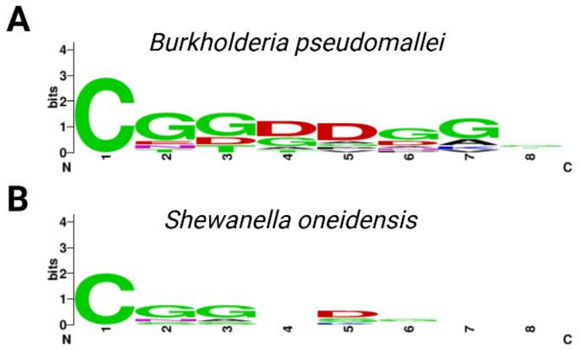

Supplemental Figure 12. Sorting motif is conserved across GspN containing T2SS. Proteomic datasets determining *gspD* dependent secreted proteins from *B. pseudomallei* strain 688 (**A**) and *S. oneidensis* strain MR-1 (**B**) were mined for lipoproteins and sorting motifs were generated for respective strain starting at the P1' residue relative to the signal peptidase II cleavage site.

Table S1. Putative inner membrane proteins from G414.

Protein	Sorting Motif
RE180_01550	**C**DSRIDAV
RE180_03225	**C**NQQEVPK
RE180_03575	**C**ILIFSAY
RE180_07055	**C**QVVNVKQ
RE180_08740	**C**DSKEVAQ
RE180_10800	**C**GAVYSAI
RE180_10945	**C**GGDMVLL
RE180_11055	**C**GKQQNEP
RE180_11805	**C**NKQPAQT
RE180_16525	**C**QNMSPSD
RE180_16530	**C**QNASPES
RE180_17100	**C**TSLGPNS

Bold indicates predicted P1’ cysteine residue relative to signal peptidase II cleavage site.

Table S2. Putative outer membrane proteins from G414.

Protein	Sorting Motif
RE180_02600	**C**LSFGPLK
RE180_02925	**C**SPVIFAE
RE180_03640	**C**QSMRGPE
RE180_04385	**C**ASRKPAT
RE180_05035	**C**ASKPQIN
RE180_05700	**C**SLAPEYQ
RE180_07535	**C**SGSIQSS
RE180_08655	**C**WAMYEVS
RE180_08730	**C**VNMQAPQ
RE180_11885	**C**SSTPQSA
RE180_12500	**C**NKHENKT
RE180_13675	**C**SIFGVYK
RE180_13725	**C**QTTGNNL
RE180_13950	**C**SSNPSKK
RE180_15305	**C**AAVVKTP
RE180_15475	**C**QTSQTVK
RE180_17745	**C**AVTSGLQ

Bold indicates predicted P1’ cysteine residue relative to signal peptidase II cleavage site.

Table S3. Putative T2SS dependent lipoproteins from *Burkholderia pseudomallei*.

Protein	Sorting Motif
BURPS668_A2859	**C**GGDDGAG
BURPS668_3144	**C**GGDDGGG
BURPS668_3846	**C**GGGGDGG
BURPS668_3613	**C**GGDDGGT
BURPS668_3884	**C**ETAVPAA
BURPS668_3613	**C**GGDDGGT
BURPS668_3454	**C**NGDDAGS
BURPS668_A1338	**C**GDGANGE
BURPS668_A2630	**C**GDGDGVV
BURPS668_2270	**C**TTTPDKP

Bold indicates predicted P1' cysteine residue relative to signal peptidase II cleavage site.

Table S4. Putative T2SS dependent lipoproteins from *Shewanella oneidensis*.

Protein	Sorting Motif
SO_1429	**C**NSGSSDV
SO_1778	**C**GGSDGNN
SO_1779	**C**GGSDGKD
SO_0404	**C**GAGDEPY
SO_A0112	**C**GGDKGFL
SO_A0110	**C**SGESRNN

Bold indicates predicted P1' cysteine residue relative to signal peptidase II cleavage site.

Table S5. Plasmids and primers used in study.

Strain/plasmid	Description	Fwd Primer 5' to 3'	Rev Primer 5' to 3'
G414	Clinical isolate from University of Michigan		
Δ*gspD*	G414 with markerless, in frame deletion of *gspD* (2)		
Δ*gspN*	G414 with markerless, in frame deletion of *gspN* (2)		
gspN+	Complemented Δ*gspN* strain with *gspN* reintroduced in genome in the intergenic region upstream of the *gspN-gspD* operon	Upstream: gtcgacaaaaagtaaatttatagcgttattc<br>Downstream: ttattacaaggtggtaactaattttatatgaagtgaattggcg	Upstream: ttgcttagactttttcttcataagcaatcgggcttg<br>Downstream: cccgggtttcctaactcttatggtgacg
MC1061	*E. coli* cloning strain (3)		
pRK2013	Helper Strain, (4)		
SY327λpir	Helper strain (5)		
KPRR1	(6)		
KPRR1 *pulA*::tn	(6)		
KPRR1 *pulN*::tn	(6)		
pSC-B-Amp/Kan	Cloning vector (Agilent)		
pMMB67	Expression vector (7)	tggctgtgcaggtcgtaaatcac	tactcaggagagcgttcaccgacaaacaac
pCVD442	Suicide vector (5)		
pInvL	pMMB67, C-terminal 6-his	atcttatacctttaggaaagattgggg	ctgtagttgccgttgtattaccatttgaacagtttggatc
pCpaA	(2)		
pBlac	(8)		
pSP$^{\beta lac}$-InvL	InvL with the SP from β-lactamse	ttttgccttcctgtttttgctggcgggggaggcggtgatg	accgcctcccccgccagcaaaaacaggaaggcaaaatgc
pSP^{LirL}-InvL	C-terminal 6-his	Tgttctaagaaagaagaagctccatattaccaaaatggttcatcaaataac	ATTTGATGAACCATTTTGGTAATAtggagcttcttctttcttagaacaacc
pSP^{Pal}-InvL	InvL with SP from Pal	aagtcgtaagccagcaggctattaccaaaatggttcatc	accattttggtaatagcctgctggcttacgacttgc
pFilF	C-terminal 6-his	tttcggtattacgttaaaaataagtaggg	ttaatggtgatgatggtgatgagacttagttttaattgctttacaagctgg
p083535	C-terminal 6-his	tgcgtattttttatgcatcttaattcatc	ttaatggtgatgatggtgatgtttaatgactttaatacgagtcgtagcc
p10730	C-terminal 6-his	aaattggatatgatgagcgcgtaatactttaagc	ttaatggtgatgatggtgatgttgttcttttacttttggaaaagcttgattaaagtc
pLirL	C-terminal 6-his	aatctagagtgtagattaatggtggtgatggtggtg	tctgcagaatctagagtgtagattaatggtggtgatggtggtg
pPal	C-terminal 6-his	tttttattccattgatggagatg	ttaatggtgatgatggtgatgttttaatagaggaggaaccgcttc
pSP^{InvL}-Pal	Pal with SP from InvL	gggaggcggtgatacaacggcaactacaggtac	ctgtagttgccgttgtatcaccgcctccccc
pPulN	pMMB67, from KPRR1	atctgctgctggagcg	gataacgcggctagtgtc
pGspN	(2)		

Supplemental References

1. J. Abramson *et al.*, Accurate structure prediction of biomolecular interactions with AlphaFold 3. *Nature* **630**, 493-500 (2024).
2. T. L. Johnson, U. Waack, S. Smith, H. Mobley, M. Sandkvist, Acinetobacter baumannii Is Dependent on the Type II Secretion System and Its Substrate LipA for Lipid Utilization and In Vivo Fitness. *J Bacteriol* **198**, 711-719 (2015).
3. C. T. Lutz, W. C. Hollifield, B. Seed, J. M. Davie, H. V. Huang, Syrinx 2A: an improved lambda phage vector designed for screening DNA libraries by recombination in vivo. *Proc Natl Acad Sci U S A* **84**, 4379-4383 (1987).
4. V. C. Knauf, E. W. Nester, Wide host range cloning vectors: a cosmid clone bank of an Agrobacterium Ti plasmid. *Plasmid* **8**, 45-54 (1982).
5. M. S. Donnenberg, J. B. Kaper, Construction of an eae deletion mutant of enteropathogenic Escherichia coli by using a positive-selection suicide vector. *Infect Immun* **59**, 4310-4317 (1991).
6. C. L. Holmes *et al.*, Klebsiella pneumoniae causes bacteremia using factors that mediate tissue-specific fitness and resistance to oxidative stress. *PLoS Pathog* **19**, e1011233 (2023).
7. J. P. Furste *et al.*, Molecular cloning of the plasmid RP4 primase region in a multi-host-range tacP expression vector. *Gene* **48**, 119-131 (1986).
8. A. Shannon *et al.*, The PDZ domain of EpsC is required for extracellular secretion of VesB by the Type II secretion system in Vibrio cholerae. *J Bacteriol* 10.1128/jb.00144-25, e0014425 (2025).
